# Cytotoxic Effects on HL-60 Cells of Myosin Light Chain Kinase Inhibitor ML-7 Alone and in Combination with Flavonoids

**DOI:** 10.5487/TR.2009.25.4.181

**Published:** 2009-12-30

**Authors:** Joong Won Lee, Yang Jee Kim, Young Joo Choi, Hae Dong Woo, Gye Eun Kim, Tae Kyung Ha, Young Hyun Lee, Hai Won Chung

**Affiliations:** School of Public Health and Institute of Health and Environment, Seoul National University, 28 Yongon-dong, Jongno-gu, Seoul, 110-460 Korea

**Keywords:** Myosin light chain kinase inhibitor, 1-(5-iodonaphthalene-1-sulfonyl)-1H-hexahydro-1,4-diazapine hydrochloride (ML-7), Apoptosis, DNA damage, Flavonoids, HL-60 cells

## Abstract

Uncontrolled cell growth and increased cell proliferation are major features of cancer that are dependent on the stable structure and dynamics of the cytoskeleton. Since stable cytoskeleton structure and dynamics are partly regulated by myosin light chain kinase (MLCK), many current studies focused on MLCK inhibition as a chemotherapeutic target. As a potent and selective MLCK inhibitor, ML-7 [1-(5-iodonaphthalene-1-sulfonyl)-1H-hexahydro-1,4-diazapine hydrochloride] is a promising candidate for an anticancer agent, which would induce apoptosis as well as prevents invasion and metastasis in certain types of cancer cells. This study assessed cytotoxic effects of ML-7 against HL-60 cells and therapeutic efficacy of ML-7 as a potential antileukemia agent. Trypan-blue exclusion assays showed dose- and time- dependent decreases in ML-7 treated HL-60 cells (p < 0.05). Comet assays revealed a significant increase in DNA damage in HL-60 cells after treatment with 40 µM ML-7 for 2 h. Sub-G1 fractions, analyzed by flow cytometry increased in a dose-dependent manner, suggesting that ML-7 can induce apoptotic cell death in HL-60 cells. ML-7 was selectively cytotoxic towards HL-60 cells; not affecting normal human lymphocytes. That selective effect makes it a promising potential anti-leukemia agent. In addition, anticancer efficacy of ML-7 in combination with flavonoids (genistein or quercetin) or anticancer drugs (cisplatin or AraC) against HL-60 cells was assessed. Combination of ML-7 with flavonoids increased the anticancer effect of ML-7 to a greater extent than combination with the anticancer drugs. This implies that ML-7 in combination with flavonoids could increase the efficacy of anticancer treatment, while avoiding side effects cansed by conventional anticancer drug-containing combination chemotherapy.

## INTRODUCTION

Carcinogenesis and cancer cell growth are dependent on rapid cell division. For rapid cancer cell division, stable cytoskeletal structures are needed and dynamic organization of the actin cytoskeleton has to be supported. Therefore, destabilizing the cytoskeleton by inhibiting the proteins that take part in cytoskeletal change is a novel prospective target when treating cancer (Giganti and Friederich, 2003; Jordan and Wilson, 1998; Jordan and Wilson, 2004).

Cytoskeletal organization and structural stiffness are primarily modulated by forces that are generated by actin-myosin II interaction (Adelstein, 1983; Mahajan *et al.*, 1989; deLanerolle and Paul, 1991). Actin-myosin II interaction is regulated by phospho- and dephosphorylation of the 20 kDa myosin II regulatory light chain (MLC20) which is catalyzed by myosin light chain kinase (MLCK). MLCK, a $Ca^{2+}$-calmodulin dependent multi-functional enzyme, regulates smooth muscle (Kamm and Stull, 1985) and non-muscle cell contractions (Bresnick, 1999) through myosin activation, and plays a key role in a variety of cellular processes, including cell motility (Clarke and Spudich, 1977; Wilson, 1991; Jay, 1995), endothelial (Verin *et al.*, 2001; Wysolmerski and Lagunoff, 1990; Wysolmerski and Lagunoff, 1991) and epithelial (Ostrow, 1994; Kolodney and Elson, 1993; Shen *et al.*, 2006) barrier function, and cytokinesis (Adelstein, 1983; Yamakita, 1994; Jana *et al.*, 2006). Consequently, dephosphorylation of MLC20 by inhibition of MLCK induces apoptosis of smooth muscle and epithelial cancer cells and regulates cell death (Fazal *et al.*, 2005; Jin *et al.*, 2001; Connell and Helfman, 2006). It also inhibits solid and epithelial cancer invasions and metastases (Thomas *et al.*, 2002; Kaneko *et al.*, 2002; Gu *et al.*, 2006; Zhou *et al.*, 2008; Suzuki *et al.*, 2004).

Recently, therefore, inhibition of MLCK has generated interest as a therapeutic target, and ML-7 [1-(5-iodonaphthalene-1-sulfonyl)-1H-hexahydro-1,4-diazapine hydrochloride], a potent and selective inhibitor of MLCK (Fenteany and Zhu, 2003), has become a promising anticancer drug candidate. However, several recent studies were limited in their assessment of ML-7 against invasion and metastasis of solid and epithelial cancer cells through regulation of one of the cytoskeleton's functions - cell motility.

To overcome those limitations, first (*1*) the cytotoxic effects of ML-7 on HL-60 promyelocytic leukemia cell line were investigated. Conventional anticancer drugs result in high toxicity to normal cells during cancer chemotherapy and may lead to the development of primary and secondary drug resistance in cancer cells, thereby limiting clinical success during cancer chemotherapy (Chabner and Roberts Jr., 2005; Goldie, 2001). Secondly (*2*), and with those observations in mind, we assessed the potentiality of ML-7 as a potential anti-leukemia agent by examining whether it had selective cytotoxic tendencies, toward HL-60 cells, but not toward normal cells. In addition, recent reports show that combination chemotherapies for cancer treatment can be superior to single-agent therapies, and that naturally occurring dietary supplements with known anti-cancer activity and with the capacity to reduce systemic toxicity of chemotherapeutic agents, can be used as agents in combination chemotherapy (Hortobagyi, 2001). Third (*3*), and based on those observations, we assessed ML-7 as a potential anti-leukemia agent by investigating the combined treatment of ML-7 with anticancer drugs and with flavonoids, to explore possible increased anticancer effects. The results showed that, clinically, ML-7 has potential as an anticancer agent and, if combined with flavonoids rather than conventional anticancer drugs, may reduce the side-effects of combination chemotherapy treatment.

## MATERIALS AND METHODS

***Cell isolation and cell culture.*** Separation of peripheral blood lymphocytes was carried out under sterile conditions on Ficoll-Paque gradients by the method of Boyum (Boyum, 1976). Peripheral blood mononuclear cells (PBMCs) were obtained from consenting healthy adult donors. The PBMCs were isolated by buoyant density centrifugation at 400 ×g, using Ficoll-Paque™ (Amersham Biosciences, Uppsala, Sweden), removed from the interface, washed twice and resuspended in RPMI-1640 (Gibco, Paisley, UK), that was supplemented with 10% fetal bovine serum (FBS) (Gibco, Paisley, UK), and 100 U/ml penicillin/streptomycin (Sigma, St. Louis, MO, USA). The cultures were stimulated with 1% phytohemagglutinin (PHA) (Gibco, Paisley, UK). Human leukemia cell line HL-60 cells (KCLB10240, Seoul, Korea) were cultured in RPMI-1640 supplemented with 10% fetal bovine serum and 100 U/ml penicillin/streptomycin. Both cells were kept in a 37°C humidified atmosphere with 5% $CO_2$.

***ML-7 treatment.*** The MLCK inhibitor ML-7 (catalog no. I-2764, Sigma-Aldrich) was dissolved in DMSO at the concentration of 25 mM as stock solution. Before the treatment of the ML-7, cells were harvested and seeded into well plates. And then cells were harvested after treatment with various times and concentrations of ML-7.

***Cell viability assay.*** Cell viability was measured using trypan blue exclusion assay. Cells were grown at a density of $1 \times 10^6$ cells/ml for trypan blue exclusion assay. Cells were cultured for 0 to 24 h after treatment with various concentration of ML-7. Viable cells were analyzed by determining their ability to exclude the dye.

***Single-cell gel electrophoresis (Comet assay).*** The single-cell gel electrophoresis (Comet assay) was used to assess DNA damage such as DNA strand breaks and abasic sites as described by Singh et al (Singh *et al.*, 1988). After ML-7 treatment for 4 h, cells were mixed with 85 μl of 0.5% low-melting agarose (LMA) at 37°C and pipetted onto the first agarose layer. Another 85 μl of 0.5% LMA was layered top. The cells were then lysed with freshly prepared cold lysis solution (2.5 M NaCl, 100 mM $Na_2$ EDTA, 10 mM Tris; pH 10 with 1% Triton X-100) at 4°C for 1 h. The slides were placed on Horizontal gel electrophoresis unit, and the slides were left in the alkaline Electrophoresis buffer (1 mM $Na_2$-EDTA, 300 mM NaOH, pH 13) for 30 min for the DNA to unwind. The slides were neutralized 3 times and fixed by absolute ethanol. Sixty randomly selected cells per slides were examined with the KOMET 5.5 image analysis system. Olive tail moment (OTM) was used as a measure of DNA damage. In each cell, the OTM was measured under a microscope (Nikon, Tokyo, Japan) equipped with an excitation filter of 515 to 560 nm and a barrier filter of 590 nm.

***Detection and Quantification of apoptosis.*** After ML-7 treatment for various lengths of time, Apoptotic cells were identified by using Micronuclei assay. The cells were collected and treated with 0.075 M KCl hypotonic solution for 1 min and fixed in the mixture of methanol : acetic acid (3 : 1). Air-dried preparations were made and stained by Giemsa staining. The nuclear morphology of the cells was observed under the optical microscope (final magnification × 400). Apoptosis was quantificated by propidium iodide (PI) staining and fluorescence-activated cell sorting (FACS) analysis (Nicoletti *et al.*, 1991). In brief, cells were harvested, washed twice with PBS and fixed in cold 70% ethanol and stored at -20°C until analysis. Before flow cytometry analysis, the fixed cells were washed twice and DNA was stained with 50 μg/ml propidium iodide (PI) (Sigma, St. Louis, MO, USA) solution for 20 min at room temperature. DNA contents of cells were then quantificated

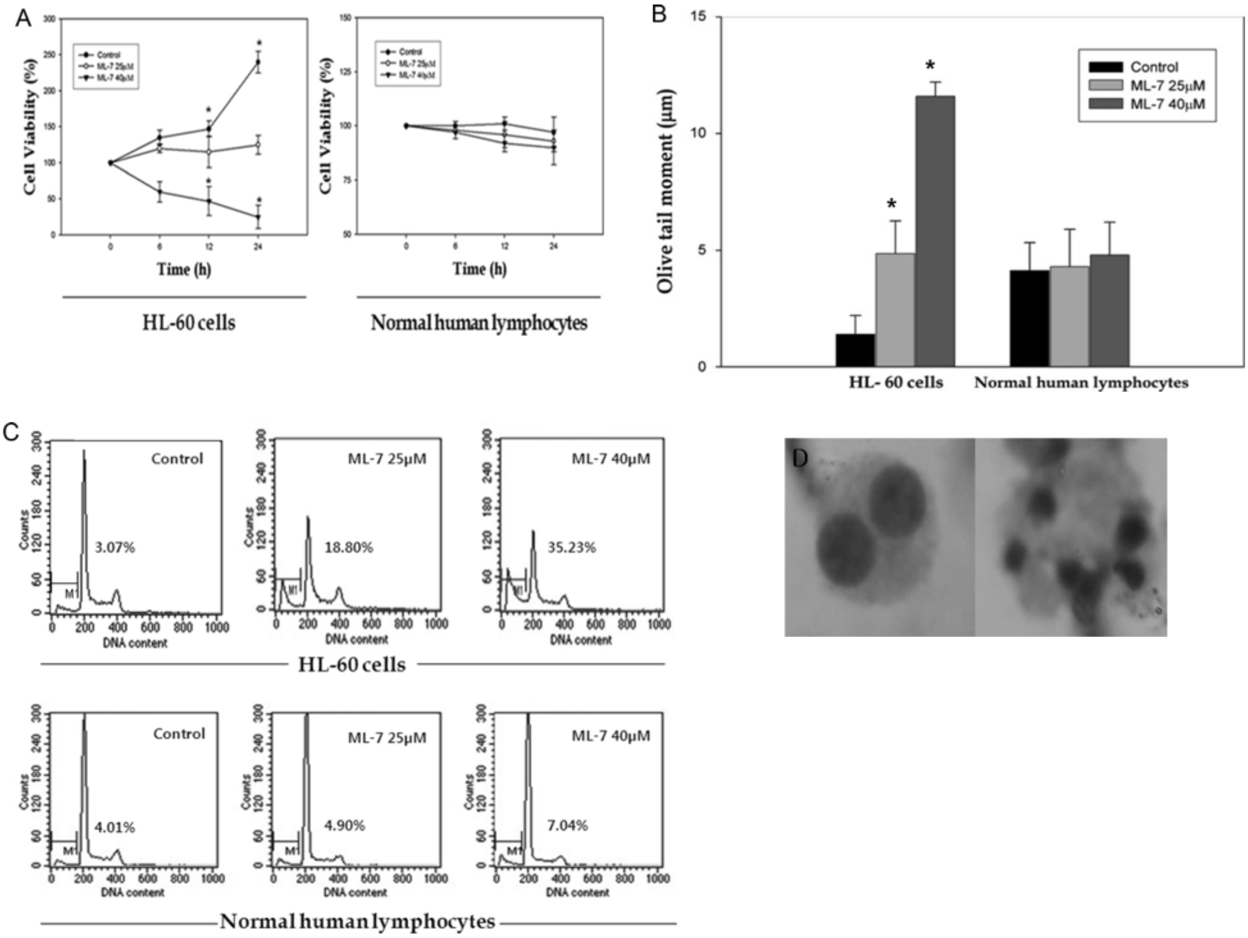

**Fig. 1.** Selective cytotoxic effect of ML-7 on HL-60 cells and normal human lymphocytes. (A) HL-60 cells were treated with the indicated concentrations of ML-7 for 0~24 h and trypan blue exclusion assays were used to measure cell viability. Viability was defined as the percentage of viable cells within the total number of cells. Human lymphocytes were activated by treatment with PHA. Human lymphocytes were exposed to different concentrations of ML-7 for 0~24 h, and their viability was determined using the trypan-blue exclusion method. Values are means ± S.E.M. ($n = 3$); * indicates $P < 0.05$ significance of differences compared to untreated controls (Student's *t*-test). (B) The alkaline comet assay was used to determine DNA single-strand breaks, and the Olive tail moment was used as an index of damage [(tail mean head mean) × Tail%DNA/100]; *indicates $P < 0.05$ significance of differences compared to untreated controls (Mann-Whitney U test). (C) Analysis of apoptotic cell death in HL-60 cells and normal human lymphocytes treated with ML-7 determined by using PI (propidium iodide) staining, as measured by flow cytometry. HL-60 cells and normal human lymphocytes were treated with control, 25, and 40 μM of ML-7 at 37°C for 9 h. The results from one representative experiment are shown; similar patterns were revealed in three separate experiments. (D) HL-60 cells were treated with ML-7 (40 μM) for 6 h, and nuclear morphologic changes in these treated cells were evaluated in using giemsa-staining. Compared with untreated control groups (left), treated group (right) had much more cell with the apoptotic characteristics.

by Fluorescent Activated Cell Sorter using CellQuest Pro software.

***Statistic analyses.*** Statistical analyses of data were performed using SAS software version 8.1 for Windows (SAS Institute, Inc., Cary, NC, USA). Olive tail moments induced by ML-7 were compared with untreated controls using the Mann-Whitney U test. Student's *t*-test was used for analysis of cell viability. Data are expressed as means ± SEM, and $P < 0.05$ was considered statistically significant.

## RESULTS

***Selective cytotoxic effects of ML-7 on HL-60 cells and normal human lymphocytes.*** To determine the cytotoxic effects of ML-7 on HL-60 cells and normal human lymphocytes, our analysis was based on the trypan blue dye exclusion assay. Figure 1A shows the viability of HL-60 cells and normal human lymphocytes after incubation with various concentrations of ML-7 for 6, 12, and 24 h. ML-7 treated HL-60 cells exhibited significant dose- and time-dependent decreases in cell viability. However, unlike the HL-60 cells, normal human lymphocytes under the same conditions did not show a significant decrease in cell viability (Fig. 1A). We also observed selective DNA-damaging effects of ML-7 on HL-60 cells and on normal human lymphocytes using comet assays to quantitate DNA damage such as single strain DNA breaks and abasic sites. Figure 1B shows the effect of ML-7 on DNA damage. HL-60 cells exposed to ML-7 had a significantly greater Olive tail moment (OTM) values than those not exposed ($P < 0.05$, Mann-Whitney U test), while normal human lymphocytes under the same conditions did not show significant OTM differences from the controls.

Following detection of selective cytotoxic effects of ML-7, HL-60 cells and normal human lymphocytes were collected and apoptosis was quantitated. As shown in Fig. 1C, apoptotic cell death was induced by treatments of 25 μM and 40 μM of ML-7 for 9 h in HL-60 cells, resulting in apoptotic cell proportions of 18.80% and 35.23%, respectively, compared with only 3.07% in the untreated HL-60 controls. In contrast, human lymphocytes with 25 μM and 40 μM of ML-7 for 9 h did not exhibit a change in the proportions of apoptotic cells (4.90%, 7.04%) compared with that in the untreated control (4.01%). We also observed induction of apoptosis after giemsa-staining of the fixed cells showing apoptotic bodies after 6 h of ML-7 exposure in HL-60 cells (Fig. 1D).

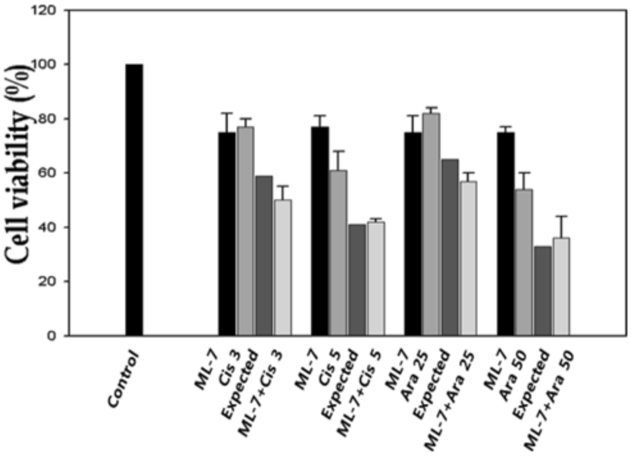

**Fig. 2.** Combination effects of ML-7 with a conventional anticancer drug (cisplatin, Ara-C) on HL-60 cells. Cells were treated with 3, 5 μM cisplatin or 25, 50 μM Ara-C in the presence of 25 μM ML-7 for 9 h. Trypan blue exclusion assays were used to measure cell viability. Cell viability was expressed relative to untreated controls (designated 100%). Values are means ± S.E.M. ($n = 3$). Expected = cell viability of ML-7 treatment only + Cell viability of cisplatin or Ara-c treatment only.

***Combination effects of ML-7 with conventional anticancer drugs (cisplatin, Ara-C) on HL-60 cells.*** To investigate the combination effect of ML-7 + cisplatin or ML-7 + Ara-C, HL-60 cells were co-treated with cisplatin (3 μM, 5 μM) or Ara-C (25 μM, 50 μM) in the presence of ML-7 (25 μM) for 9 h. Figure 2 shows the cell viabilities of HL-60 cells exposed to 25 μM of ML-7 and various concentrations of cisplatin and Ara-C. To verify whether the combined effects of ML-7 and conventional anticancer drugs were additive or synergistic, we compared the differences between expected values and the combined treatments. Expected values are following as: Expected = cell viability of ML-7 treatment only + cell viability of conventional anticancer treatment only. ML-7-only treatment cells exhibited 75% cell viability. 77% and 61% cell viability were shown in 3, 5 μM of cisplatin treatment and 82% and 54% cell viability were observed in 24, 50 μM of Ara-C treatment, respectively (control designated as 100% viability).

Compared the differences between expected values and combined treatments (ML-7 + 3 μM, + 5 μM of cisplatin treatment, ML-7 + 25 μM, + 50 μM of Ara-C treatment), the combination of ML-7 with these two anticancer drugs, produced an additive treatment efficacy. However, statistical significance of the apparent synergistic effect was not assessed.

***Combination effects of ML-7 with flavonoids (genistein, quercetin) on HL-60 cells.*** To investigate the effect on HL-60 cells of two combinations (ML-7 with genistein and ML-7 with Quercetin), HL-60 cells were treated with genistein (3 μg/ml, 5 μg/ml) and quercetin (5 μM, 10 μM) in the presence of ML-7 (25 μM) for 9 h. The ML-7-only treatment resulted in 75% cell viability, while genistein-only (3 μg/ml, 5 μg/ml) treatments showed 97, 90% cell viability, respectively, and quercetin-only (5 μM, 10 μM) treatments showed 98, 86% cell viability, respectively. Although neither genistein nor quercetin alone showed cytotoxic activity, ML-7 + genistein and ML-7 + quercetin treatments resulted in increased anticancer effects of ML-7 compared with expected values. These results indicate that there is an interaction between these flavonoids and ML-7, which appeared to produce a synergistic cytotoxic effect (Fig. 3A). In addition, fluoresence activated cell sorting (FACS) analysis also demonstrated a synergistic effect of ML-7 + genistein or ML-7 + quercetin against HL-60 cells (Fig. 3B).

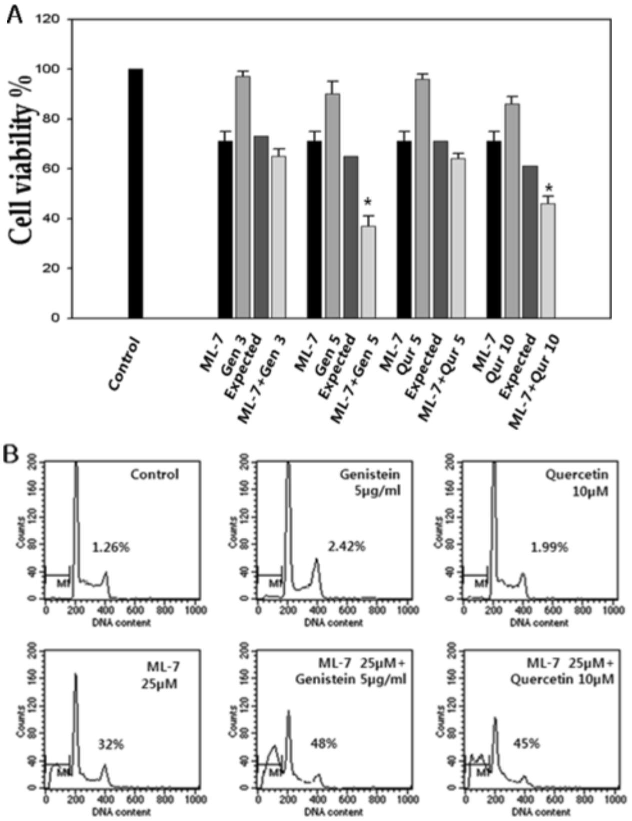

**Fig. 3.** Synergistic effects of genistein or quercetin on ML-7 induced apoptosis in HL-60 cells. (A) Cells were treated with 3, 5 μg/ml genistein or 5, 10 μM quercetin in the presence of 25 μM ML-7 for 9 h. The trypan blue exclusion assay was used to measure cell viability. Cell viability was expressed relative to untreated controls (designated 100%). Values are means ± S.E.M. ($n$ = 3); * indicates $P < 0.05$ significance of differences from expected values (Student's $t$-test). Expected = cell viability of ML-7 treatment only + cell viability of genistein or quercetin treatment only. (B) Analysis of apoptotic cell death in HL-60 co-treated with 25 μM ML-7 and 5 μg/ml genistein or 10 μM quercetin were determined using PI (propidium iodide) staining, as measured by flow cytometry. The results from one representative experiment are shown; similar patterns were revealed in three separate experiments.

## DISCUSSION

The cytoskeleton has importance in determining whether cells live or die, and destabilizing the cytoskeleton may be a good way to induce apoptosis in cancer cells. The organization and stiffness of the cytoskeleton are determined in large part by the forces generated by actin-myosin II interactions (Adelstein, 1983; Mahajan *et al.*, 1989; De Lanerolle and Paul, 1991), which are regulated by the phosphorylation of serine 19 of the 20-kDa light chain of myosin II (MLC20) that is catalyzed by myosin light chain kinase (MLCK). The pharmacological agent ML-7, which selectively inhibits MLCK interrupts actin-myosin II interactions, leading to destabilization of the actin cytoskeleton, and resulting in the induction of apoptosis (Fazal *et al.*, 2005; Jin *et al.*, 2001; Connell and Helfman, 2006). Despite the fact that many reports have demonstrated that MLCK inhibition by ML-7 leads to apoptosis in various cell types, its cytotoxic effect on leukemia cells have not yet been elucidated.

In our experiment, we evaluated the cytotoxic effect of ML-7 on the HL-60 leukemic cell line. We found that the exposure of ML-7 to HL-60 cells resulted in a dose- and time-dependent decrease in cell viability (Fig. 1A) and induced apoptotic cell death of HL-60 cells, as demonstrated by the increase of sub-G1 fraction (Fig. 1C). Jasplakinolide, like ML-7, disrupts the actin cytoskeleton through the induction of actin polymerization and/or the inhibition of the depolymerization of actin filaments, and not only inhibits cell growth by interrupting actin cytoskeleton dynamics during cytoskeleton remodeling (Bai *et al.*, 2001), but also leads to apoptosis accompanied by an increase in caspase-3 activation (Posey and Bierer, 1999; Rao *et al.*, 1999; Odaka *et al.*, 2000). Our results are in accordance with the study that ML-7 induces apoptosis, and implies that the actin cytoskeleton plays a central role in apoptotic cell death against HL-60 cells. Although there are no studies of the mechanism of actin cytoskeleton involvement in apoptosis in mammalian cells, there is such evidence in studies using yeast cells. The crucial findings in yeast cell studies (Gourlay and Ayscough, 2003, 2005, 2006; Farah and Amberg, 2007) are that the inhibition of proteins related to cytoskeletal organization induces alteration of actin dynamics and leads to an increase in reactive oxygen species (ROS) levels (e.g., $H_2O_2$, hydroxyl radical, and superoxide), which are intimately related with cell death induction. Therefore, we speculated that when actin dynamics are altered by ML-7, there are ROS generated that might be involved in HL-60 cell death.

We investigated not only the cytotoxic effect of ML-7 on leukemic cells, but also its potential as an anti-leukemia agent. The cytotoxicity towards leukemic cells was tested by comparing it with cytotoxicity in normal human lymphocytes obtained from healthy donors. ML-7 showed a cytotoxic effect that was selective towards leukemic cells, but it did not affect normal cells (Fig. 1A, Fig. 1B, Fig. 1C). This selective effect suggests that ML-7 has potential as a anti-leukemia agent; one that would have few toxic side effects on normal cells.

With the intention of enhancing the anticancer efficacy of ML-7 against HL-60 cells we investigated the anticancer effect of ML-7 in combination with conventional anticancer drugs (cislatin or Ara-C) and with flavonoids (genistein or quercetin). The central finding of these experiments was that the flavonoids enhanced the anticancer efficacy of ML-7 on HL-60 cells to a greater extent than that by cisplatin and Ara-C; thus, indicating a synergistic effect between ML-7 and flavonoids. Although cisplatin and Ara-C are well known cancer therapeutic agents, they cause high toxicity to normal cells during cancer chemotherapy. Furthermore, these agents lead to the development of primary and secondary drug resistance in cancer cells thereby limiting their clinical success in the cancer chemotherapy (Chabner and Roberts Jr., 2005; Goldie, 2001). In these regard, recent reports show that some combination chemotherapies produce superior cancer treatment results than single-agent therapies. Moreover, naturally occurring dietary supplements with known anti-cancer activity can be used as one of the agents in combination chemotherapy in order to reduce systemic toxicity of the chemotherapeutic agents (Hortobagyi, 2001). Flavonoids are non-toxic agents that are widely consumed as dietary supplements. Also, recent *in vitro* studies, epidemiological investigations, and human clinical trials have shown that flavonoids not only have chemo-preventive properties, but also can function as anti-proliferates, and furthermore can arrest the cell cycle, induce apoptosis and differentiation, and inhibit the angiogenesis (Nijveldt *et al.*, 2001; Galati and O'Brien, 2004). There are the several possible reasons why the treatment of HL-60 cells with ML-7 in combination with flavonoids enhanced the anticancer efficacy of ML-7. According to a recent study, flavonoids interfere with the function of actin in cytoplasm and nucleus by binding to actin, which leads to inhibition of the actin polymerization rate, transcription efficiency, and actin conformation (Bohl *et al.,* 2007). This implies that flavonoids, which have specific and high affinity to actin, act as an enhancer of ML-7 treatment by affecting an actin-related molecular mechanism in ML-7-induced cell death. According to Gu *et al.*, ML-7 in combination with etoposide enhanced the ability of ML-7 to kill cancer cells. Etoposide is a well known anticancer drug that acts by inhibiting topoisomerase II activity, and genistein and quercetin are also reported to inhibit topoisomerase II activity (Skibola and Smith, 2000; Birt *et al.*, 2001). Based on those observations when flavonoids and ML-7 are used as co-treatments, the flavonoids may enhance the ability of ML-7 to kill cancer cell by inhibiting topoisomerase II activity. It is possible that the composite actions of inhibiting topoisomerase II activity and destabilizing the actin cytoskeleton by flavonoids is toxic to cancer cells. Thus, the co-treatment with ML-7 and flavonoids could be useful to increase anticancer treatment efficacy while avoiding possible side effects related to conventional anticancer drug-containing combination chemotherapy.

In conclusion, this study shows that: 1) ML-7 induces DNA damage and apoptotic cell death in HL-60 cells; 2) ML-7 has a selective cytotoxic effect toward leukemic cells, but not toward normal cells; and 3) there is a synergistic effect on ML-7 on HL-60 cells when ML-7 treatment is combined with nontoxic, natural compound flavonoids such as genistein and quercetin. Taken together, our results suggest that ML-7 may be a promising candidate as a therapeutic anti-leukemia agent, and that its efficacy can be enhanced by flavonoid co-treatment.

## ACKNOWLEDGMENTS

This work was supported by the Brain Korea 21 Project in 2008.
